# Single Sitting Surgical Treatment of Generalized Aggressive Periodontitis Using GTR Technique and Immediate Implant Placement with 10-Year Follow-Up

**DOI:** 10.1155/2018/6194042

**Published:** 2018-01-23

**Authors:** Fatme Mouchref Hamasni, Fady El Hajj, Rima Abdallah

**Affiliations:** Department of Periodontology, Faculty of Dental Medicine, Lebanese University, Hadath, Lebanon

## Abstract

This case report exhibits a patient with generalized aggressive periodontitis who has been under maintenance for the past 12 years after being surgically treated in a single sitting and restored with dental implants. A 41-year-old systemically healthy male patient presented complaining of lower anterior teeth mobility and pain in the upper right quadrant. After clinical and radiographic examination, the upper right molars and lower anterior incisors were deemed unrestorable. Covered by doxycycline, the patient received a nonsurgical periodontal treatment. Three weeks later, teeth extraction, immediate implant placement, immediate nonloading provisional prosthesis, and a guided tissue regeneration were performed at indicated areas in a single sitting. The clinical decisions were based on patient compliance, the status of the existing periodontal tissues, and the prognosis of the remaining teeth. During the 12-year follow-up period, no residual pockets were observed and there was no exacerbation of the inflammatory condition. Marginal bone stability is present on all implants. For aggressive periodontal disease, a high risk of relapse as well as limited success and survival of dental implants should be considered. This case shows proper containment of the disease based on appropriate treatment planning and a strict maintenance program.

## 1. Introduction

In 1999, the term “aggressive periodontitis” (AgP) was introduced by the American Academy of Periodontology (AAP) to define a group of destructive periodontal diseases with a rapid progression. This definition was used to include previous terminologies of early-onset periodontitis, juvenile periodontitis, and rapidly progressive periodontitis, using “aggressive” nomenclature [1]. The emphasis was placed on the rapidity of progression, rather than on the age of onset (destruction is 2–5 times faster than in chronic periodontitis with an attachment loss of 4 to 5 micrometers per day) and perhaps also on the difficulty of maintaining it.

Lang et al. [2] classified the disease into localized and generalized forms. According to this report, the authors concluded that there are common features in both the localized and generalized forms of aggressive periodontitis. All symptoms can occur at any age in patients who follow regular dental care. A familial factor is present, and there is a possibility of self-arresting progression of attachment loss (AL) and bone loss.

Microbiological criteria were not mentioned as primary features separating chronic from aggressive periodontitis. However, for AgP, the secondary features that are generally, but not universally, present included elevated proportions of *Aggregatibacter actinomycetemcomitan* (AA) and, in some populations, *Porphyromonas gingivalis* (Pg), phagocyte abnormalities, and a hyper-responsive macrophage phenotype, including elevated levels of prostaglandin E2 and interleukin-1beta.

The following additional specific features were proposed for defining the localized and generalized forms. Localized aggressive periodontitis is usually circumpubertal onset, localized to first molar and incisor presentation with interproximal attachment loss on at least two permanent teeth (one of which is a first molar) and involving no more than two teeth other than first molars and incisors. Generalized aggressive periodontitis usually affects adult persons between 20 and 30 years of age, but patients may be older. There is generalized interproximal attachment loss affecting at least three permanent teeth other than first molars and incisors. The disease has a pronounced episodic nature of the destruction of attachment and alveolar bone with a history of relapse.

Although success in implant dentistry depends on marginal bone stability and health, patient systemic factors and susceptibility to periodontal diseases play a role in achieving long-term stability.

Many studies have shown the negative effect of previously treated and untreated periodontal disease on marginal bone stability around implants, including higher frequency of mucositis and peri-implantitis and lower success and survival rates of implants placed in patients with history of chronic or aggressive periodontitis [3–12].

Swierkot et al. [13] reported in a prospective study with a follow-up period of 5–16 years that GAgP patients had five times greater risk of implant failure, three times greater risk of mucositis, and 14 times greater risk of peri-implantitis.

This case report exhibits a 41-year-old systemically healthy male patient with GAgP who has been maintained for the past 12 years after being treated periodontally in a single sitting and restored with dental implants. He has been compliant for 10 years with supportive and maintenance therapy since the conclusion of his treatment in 2007.

## 2. Case Presentation

### 2.1. Patient History and Chief Complaint

An engineer living abroad sought a second opinion at our office in 2005. The patient's oral surgeon had previously recommended extraction and immediate implantation of all compromised teeth including any tooth with a vertical defect. He sought an alternative treatment option after being shocked that at only 41 years of age, the only treatment will be losing 16 of his teeth. The patient's chief complaint was that he is not able to bite on his front teeth and that his esthetics are compromised due too increased crown length.

### 2.2. Initial Assessment

**Figure 1:**
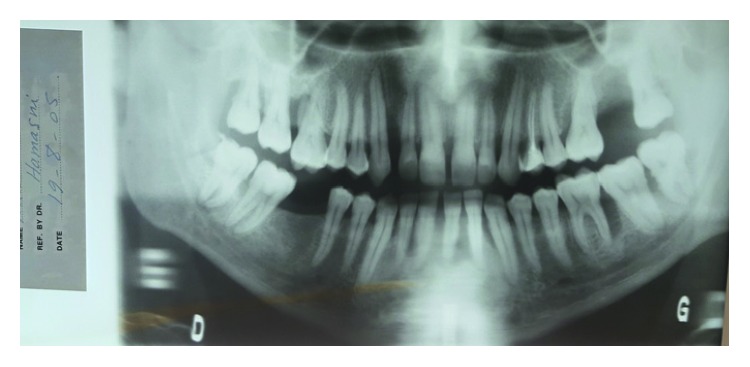
Preoperative panoramic X-ray showing generalized bone loss and missing teeth 27 and 46.

**Figure 2:**
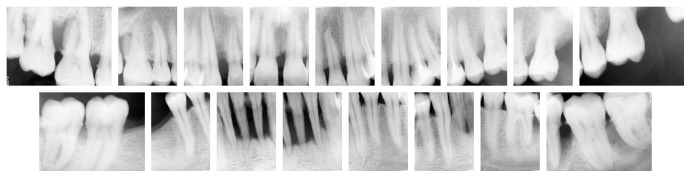
Full mouth periapical X-rays showing severe bone loss at lower anterior sextant and vertical bone loss at 13 and 37.

Diagnostics included radiographic examination (Figures 1 and 2) and a thorough clinical examination. Probing was done at the affected sites, and it was noted that there was probing depth of more than 10 mm at 11 sites and between 6 and 9 mm at 6 sites. He was missing teeth number 27 and 46.

A conventional supra gingival scaling and subgingival root planing was performed under the coverage of antibiotics having a significant action against AA (doxycycline (Doxylag)® 100 mg 2 tabs first day and 1 tab daily for 21 days) [14, 15] and a chlorohexidine mouth wash (0.1% chlorohexidine and 0.5% chlorobutanol) for a period of 14 days to assess the periodontal tissue response and to stabilize the condition. This initial assessment led to the establishment of the prognosis for the remaining teeth and identification of those that could not be treated. The patient returned for definitive treatment after 3 weeks.

### 2.3. Surgical Treatment

Upon the patients' return, teeth 18, 17, and 16 all had class III furcation involvement with grade 3 mobility; they were extracted under full thickness flap allowing visibility of a 3-wall defect of 10 mm at the mesial of tooth number 13 which was then treated with guided tissue regeneration technique using bovine xenograft bone substitute Bioss® and resorbable collagen membrane Resolute®.

At the same visit, extraction of tooth number 26 was performed as it presented with a 9 and 10 mm bone loss at the mesial and distal sites, respectively, with a class III furcation involvement.

At the lower right quadrant, tooth number 48 was extracted, and scaling and root planing was performed at the distal of tooth number 47 which presented with a wide shallow bony defect.

At the lower left quadrant, extraction of tooth number 38 was done, and the full thickness flap showed a narrow and deep bone defect until the apex of tooth number 46 distally maintaining the mesial bone peak at tooth number 47 which presented as well with a wide moderate bone defect distally. Scaling and root planing was done, due to the contained geometry of the defect on the 46; the bone substitute Bioss was used as a filling materiel, without the need for a membrane.

Teeth 32, 31, 42, and 41 were extracted and immediately replaced with 3 narrow neck SLA Straumann dental implants (3.3 × 12 mm at site 41 and 31 and 3.3 × 10 mm at site 32). The choice of cantilevering was based on the presence of a wide intrabony defect surrounding tooth number 42; immediate nonloading temporization was provided on implants number 41 and 32 to maintain the esthetic appearance.

No provisional prosthesis was delivered for molar areas, and an association of amoxicillin 500 mg and metronidazole 250 mg was prescribed 3 times a day for 8 days [16].

**Figure 3:**
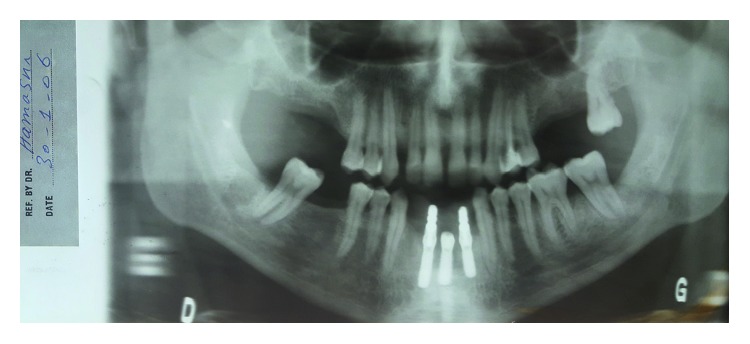
Panoramic X-ray after 3 months of first visit showing bone formation at the site of extracted molars and bone stability around Straumann mandibular implants.

**Figure 4:**
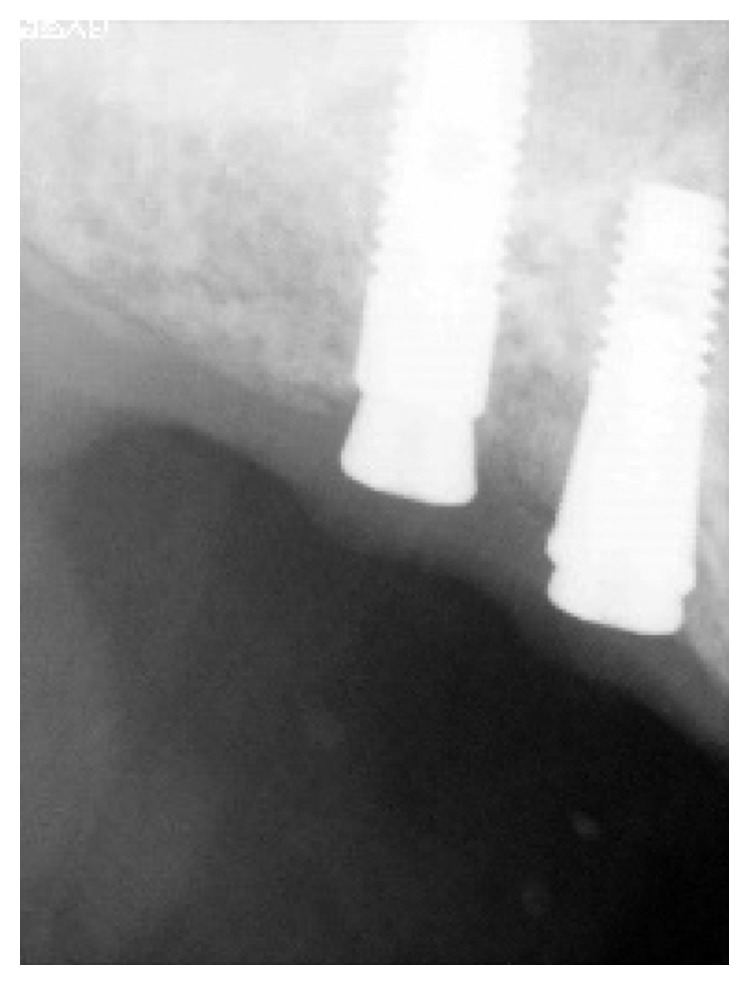
Astra^∗^ implants (4.5 × 11, 4.0 × 11) replacing teeth 16 and 17.

**Figure 5:**
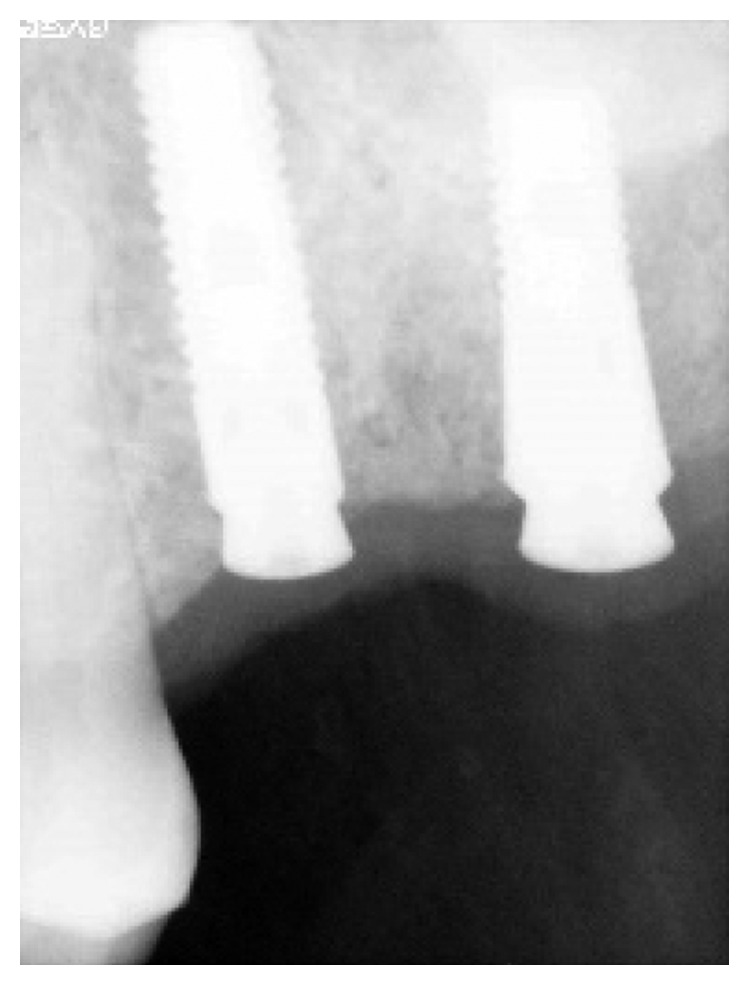
Astra^∗^ implants (4.0 × 13, 5 × 11) replacing teeth 26 and 27.

**Figure 6:**
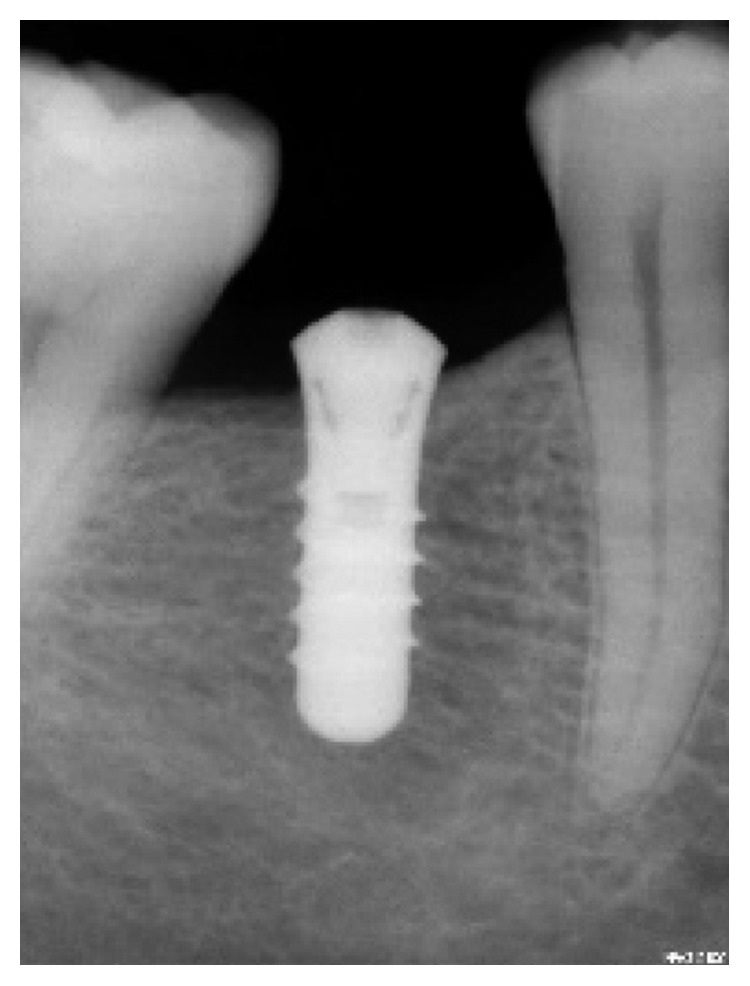
Straumann (4.1 × 10) replacing tooth 46.

Almost 5 months later, the clinical exam showed a perfect soft tissue integrity, and the radiographic evaluation revealed total bone formation at the site of extracted molars and bone stability around Straumann mandibular implants (Figure 3). Four osseospeed Astra^∗^ implants were used in the maxilla replacing the first and second molars: (4.5 × 11 mm, 4.0 × 11 mm) implants were inserted on the right side, and (4.0 × 13 mm, 5.0 × 11 mm) implants on the left side (Figures 4 and 5). One Straumann SLA tissue level implant (4.1  × 10  mm) was inserted to replace tooth 46 (Figure 6).

The patient could not return until 16 months after the implant surgery, and for the realization of final fixed prosthesis, all prosthesis were delivered within 10 days and the case was documented radiographically and clinically and follow-up maintenance program was scheduled after this visit.

### 2.4. Maintenance and Supportive Therapy

**Figure 7:**
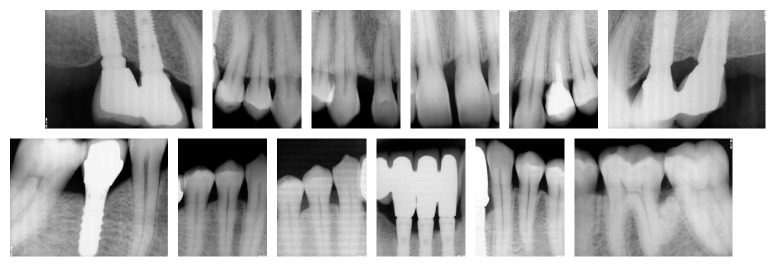
Radiographic evaluation 10 years after prosthesis insertion.

**Figure 8:**
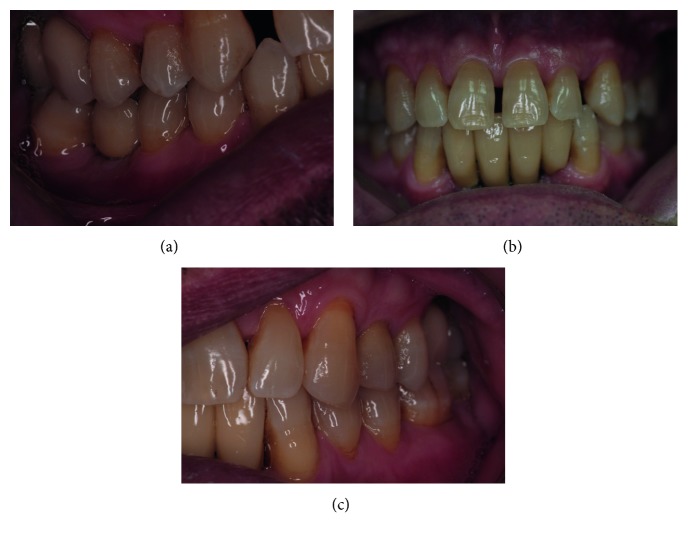
Clinical photos 12 years after periodontal surgery and 10 years after final implant prothesis.

The patient is placed on a strict maintenance schedule; prophylaxis is performed every 3 to 6 months and periapical follow-up radiographs are done every year to follow-up on the surgical sites. During the 12 year follow-up period, no residual pockets were observed, and there was no exacerbation of the inflammatory condition. Marginal bone stability is present on all implants. Since the case was done, there was no need for the adjunct use of antibacterial mouth rinses or systemic antibiotic use (Figures 7 and 8).

## 3. Discussion

The high risk of relapse as well as limited success and survival rate of dental implants is considered as a severe complication related to aggressive periodontal disease, and this case showed a perfect bone stability, after guided tissue regeneration and around implants, over 10 years after treatment without any sign of inflammation, and the stability of such results is maybe related to the strict supportive therapy program or the choice of doing full mouth surgery in one day which may assure the complete eradication of bacteria and prevent the contamination of treated areas when surgeries are usually done at variable intervals. To the best of our knowledge, this is the first case report treating surgically a case of generalized aggressive periodontitis with GTR and immediate implantation in one single day. The successful treatment is maybe related to the choice of the treatment; however, additional clinical studies with more patients are necessary in order to support this choice.
